# Diagnostic Challenge of Crohn’s Disease in a Patient with Clinically Diagnosed Pulmonary Tuberculosis: A Case Report

**DOI:** 10.1159/crg/abwag016

**Published:** 2026-09-29

**Authors:** John Paul Escama Dela Peña, Carlos Paolo D. Francisco, Marian Dimabuyu Francisco, Marianne Linley Loo Sy-Janairo

**Affiliations:** a Institute of Digestive and Liver Diseases, St. Luke’s Medical Center - Global City, Taguig City, Philippines; b Institute of Pulmonary Medicine, St. Luke’s Medical Center - Global City, Taguig City, Philippines

**Keywords:** Crohn’s disease, Pulmonary tuberculosis, Gastrointestinal tuberculosis, Case report, Ustekinumab

## Abstract

**Introduction:**

Differentiating Crohn’s disease (CD) from gastrointestinal tuberculosis remains challenging in tuberculosis-endemic regions because of overlapping clinical, radiologic, endoscopic, and histopathologic features.

**Case Presentation:**

A 21-year-old man presented with substantial unintentional weight loss without pulmonary or gastrointestinal symptoms. Chest computed tomography showed bilateral centrilobular nodules with a tree-in-bud pattern, while abdominal imaging demonstrated long-segment ileocolitis. Despite a negative tuberculosis immunoassay, pulmonary tuberculosis was diagnosed clinically, and the intestinal findings were initially attributed to possible gastrointestinal tuberculosis. Colonoscopy showed multifocal stricturing ulceration, nodularity, and friability involving the colon and rectum. Intestinal biopsy specimens demonstrated severe chronic active inflammation; *Mycobacterium tuberculosis* polymerase chain reaction and acid-fast bacillus staining were negative. During a standard 6-month antituberculosis regimen, the patient developed intermittent abdominal pain and recurrent vomiting. Repeat chest computed tomography showed resolution of the pulmonary nodules, whereas colonoscopy after 5 months demonstrated persistent longitudinal ulcers, pseudopolyps, skip areas, and stricturing from the terminal ileum to the rectum. Histopathology showed severe chronic active colitis with cryptitis, crypt abscesses, tissue eosinophilia, and rare non-necrotizing granulomas. A Crohn’s Disease Activity Index score of 229 and the overall clinicopathologic findings favored ileocolonic stricturing CD. Mesalamine and ustekinumab were initiated, with resolution of abdominal pain and vomiting, weight gain, and a decline in fecal calprotectin from 1,740 to 320 µg/g. Follow-up colonoscopy approximately 1 year later (week 48 of ustekinumab) showed resolution of the previous extensive ulceration and friability, with pseudopolyposis and ileocecal valve narrowing. Biopsies demonstrated focal mild cecal cryptitis without active inflammation in the remaining sampled colonic segments or distal terminal ileum.

**Conclusion:**

This case underscores the diagnostic challenge of distinguishing CD from gastrointestinal tuberculosis in a patient with clinically diagnosed pulmonary tuberculosis. Persistent ileocolonic inflammation despite adequate antituberculosis therapy, repeatedly negative intestinal tuberculosis studies, and endoscopic and histopathologic features favoring CD should prompt timely diagnostic reassessment and appropriate disease-specific treatment.

## Introduction

Inflammatory bowel disease (IBD), which includes Crohn’s disease (CD) and ulcerative colitis, was historically concentrated in Western populations. Over recent decades, however, its incidence has increased in several Asian regions. An analysis of Global Burden of Disease 2021 data showed that the global age-standardized incidence rate of IBD increased from 4.22 per 100,000 population in 1990 to 4.45 per 100,000 in 2021, while the corresponding age-standardized mortality and disability-adjusted life-year rates declined. East Asia demonstrated the greatest regional increase in the age-standardized incidence rate during this period [1]. Although Philippine-specific population-based epidemiologic data remain sparse, the Asia-Pacific Crohn’s and Colitis Epidemiology Study reported a mean annual IBD incidence of 0.43 per 100,000 population in its Philippine study catchment area, while the overall mean annual incidence across 13 Asia-Pacific countries or regions was 1.50 per 100,000 [2].

In TB-endemic settings, differentiating CD from gastrointestinal tuberculosis can be particularly challenging because of overlapping clinical, endoscopic, radiologic, and histopathologic features [3, 4]. Gastrointestinal tuberculosis may present with abdominal pain, weight loss, ileocolonic ulcerations, strictures, and granulomatous inflammation, findings that may also occur in CD. Microbiologic testing of intestinal tissue, including *Mycobacterium tuberculosis* polymerase chain reaction and acid-fast bacillus staining, has limited sensitivity; consequently, negative results do not exclude gastrointestinal tuberculosis. Histologic overlap is also common, although cryptitis, crypt abscesses, and architectural distortion are more suggestive of CD [4].

When an objective response to empiric anti-TB treatment is not demonstrated, early diagnostic reassessment is warranted. Follow-up endoscopy to assess mucosal healing, together with clinical, histopathologic, radiologic, and microbiologic findings, may help distinguish gastrointestinal tuberculosis from CD [4]. A multidisciplinary diagnostic approach is essential before revising the diagnosis and treatment strategy [5]. The present case illustrates these diagnostic challenges and highlights the importance of timely reevaluation in patients with presumed gastrointestinal TB who fail to improve. This case report was prepared in accordance with the CARE guidelines, and the completed CARE checklist is provided as online supplementary material (for all online suppl. material, see https://doi.org/10.1159/crg/abwag016).

### Case Presentation

We present the case of a 21-year-old male from the Philippines, with no known comorbidities or family history of tuberculosis or IBD, who was initially diagnosed with pulmonary and gastrointestinal TB but was subsequently reclassified as having CD following persistent gastrointestinal inflammation despite completion of anti-TB therapy.

### Initial Presentation and Diagnostic Workup

The patient presented with a 1-year history of unintentional weight loss, which was initially gradual but later became more rapid and pronounced (approximately 70 to 59 kg; 15.7% loss of baseline body weight). Apart from weight loss, he had no pulmonary or gastrointestinal symptoms and denied fever, night sweats, cough, dyspnea, abdominal pain, vomiting, gastrointestinal bleeding, or altered bowel habits. Physical examination showed stable vital signs, clear breath sounds, and a soft, non-tender abdomen without palpable lymphadenopathy or peripheral stigmata of chronic disease. Baseline laboratory assessment demonstrated anemia, with a hemoglobin level of 8.7 g/dL, and mild hypoalbuminemia, with a serum albumin level of 3.2 g/dL. The erythrocyte sedimentation rate was 23 mm/h, and the C-reactive protein level was 5.35 mg/L.

Cross-sectional imaging demonstrated bilateral noncalcified centrilobular pulmonary nodules with a tree-in-bud pattern, which were considered suggestive of pulmonary tuberculosis (Fig. 1a). Long-segment ileocolitis with bowel wall thickening was also noted, with gastrointestinal tuberculosis and CD considered differential diagnoses. Despite a negative tuberculosis immunoassay, pulmonary tuberculosis was diagnosed clinically based on the patient’s weight loss and chest computed tomography findings. Because of the concurrent ileocolitis and the high local prevalence of tuberculosis, gastrointestinal involvement was initially presumed.

**Fig. 1.**
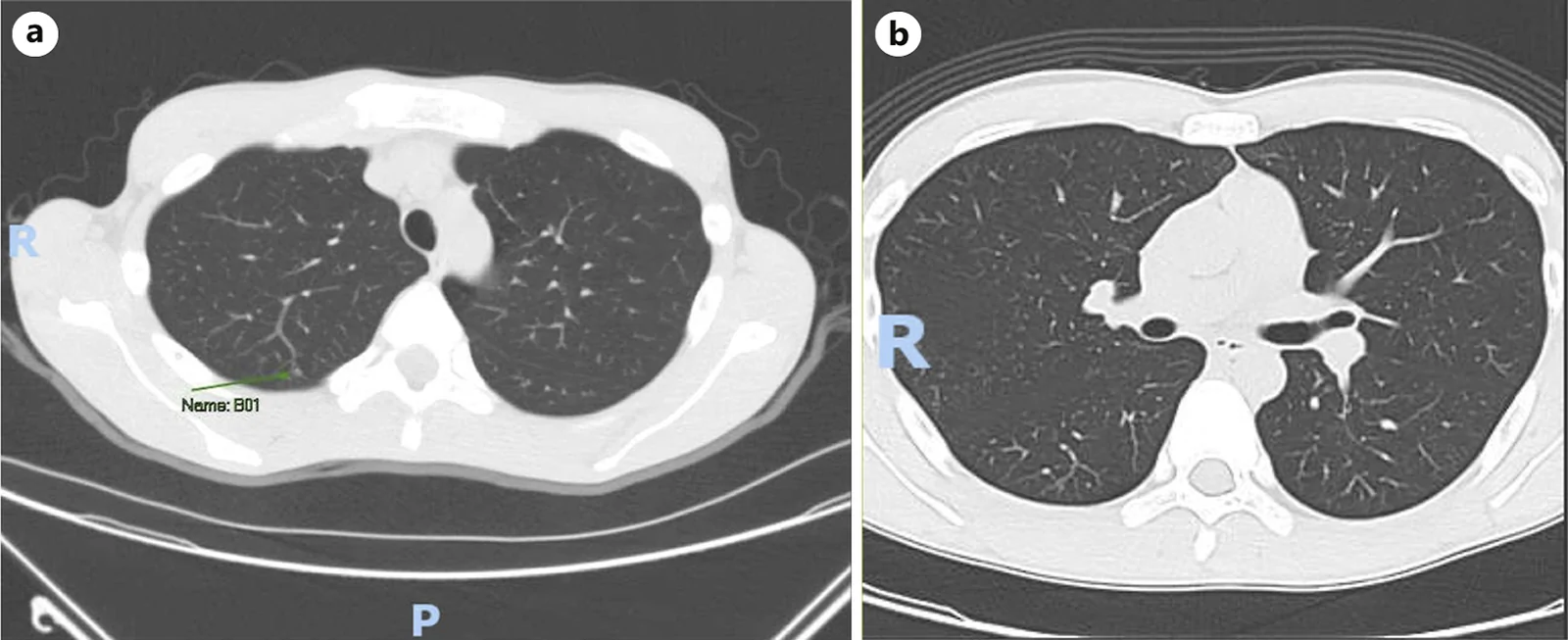
Pulmonary computed tomography findings before and after antituberculosis therapy. **a** Initial chest computed tomography showing bilateral noncalcified centrilobular nodules with a tree-in-bud pattern. **b** Follow-up chest computed tomography after 6 months of antituberculosis therapy demonstrating interval resolution of the previously observed centrilobular nodules and tree-in-bud opacities.

Because of the pulmonary imaging findings, intestinal abnormalities, and local epidemiology, he was initially treated for pulmonary tuberculosis with possible gastrointestinal involvement. The patient was started on a standard 6-month antituberculosis regimen consisting of a 2-month intensive phase with isoniazid, rifampicin, pyrazinamide, and ethambutol, followed by a 4-month continuation phase with isoniazid and rifampicin. The patient reported good adherence to the prescribed antituberculosis regimen without significant treatment interruptions. After initiation of antituberculosis therapy, he developed intermittent abdominal pain and recurrent episodes of vomiting. These new gastrointestinal symptoms further complicated the diagnostic assessment because it remained unclear whether they represented adverse effects of the antituberculosis medications, manifestations of presumed gastrointestinal tuberculosis, or an underlying IBD.

### Initial Endoscopic and Histopathologic Findings

Initial colonoscopy was advanced to the descending colon, approximately 60 cm from the anal verge, where a stricturing ulcerated segment prevented further passage of the colonoscope. The lesion consisted of irregular ulcers and prominent nodular mucosal elevations overlying markedly erythematous and friable mucosa (Fig. 2a). A second area of stricturing ulceration, nodularity, and mucosal friability was identified in the rectosigmoid colon, approximately 20 cm from the anal verge (Fig. 2b). Additional ulcers and nodular mucosal lesions were observed in the distal rectum, approximately 5 cm from the anal verge. Multiple biopsies were obtained from the descending and rectosigmoid lesions for histopathologic examination and *M. tuberculosis* polymerase chain reaction testing, with additional biopsies obtained from the distal rectal lesions.

**Fig. 2.**
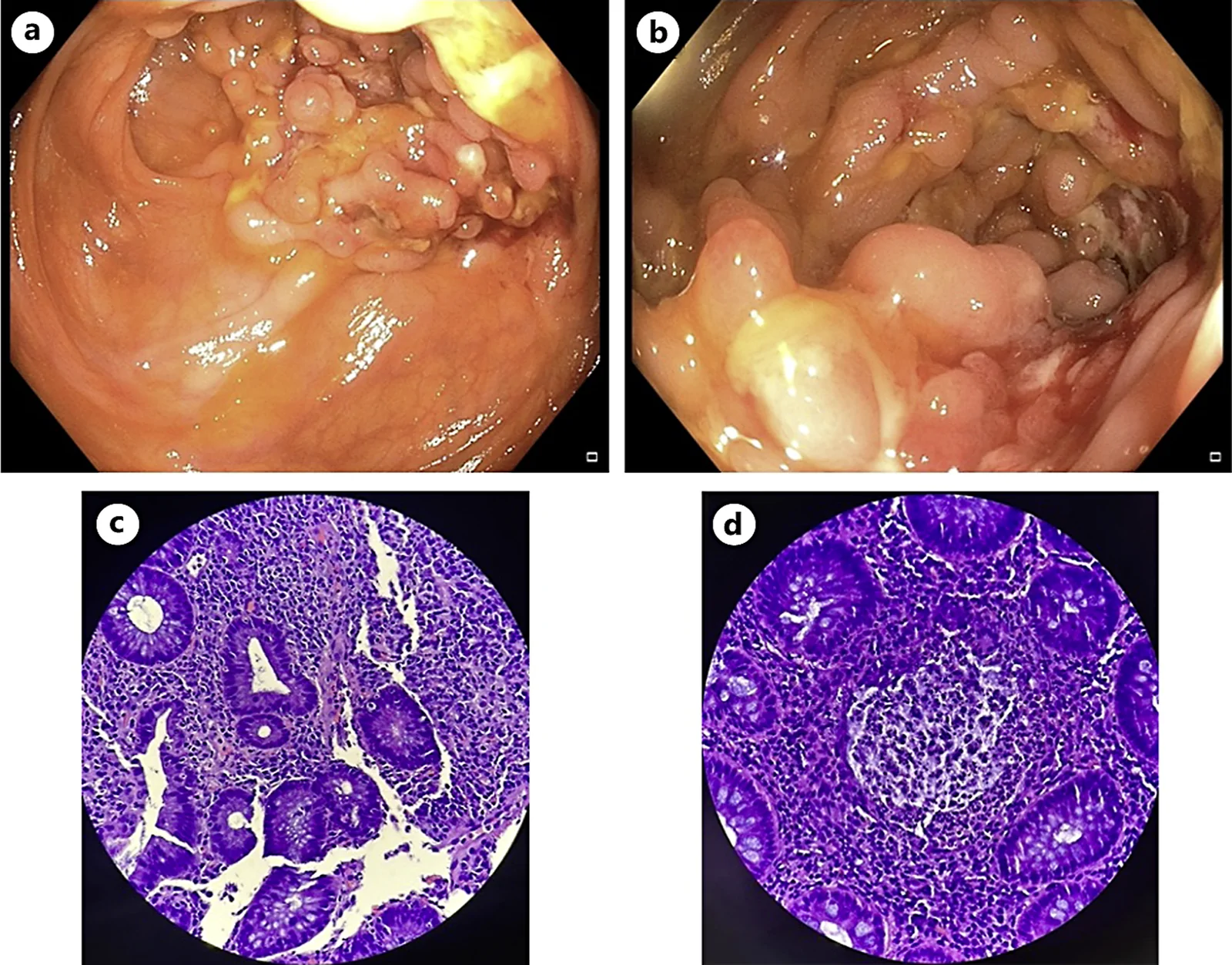
Initial colonoscopic and histopathologic findings. **a** Descending colon, approximately 60 cm from the anal verge, showing a stricturing ulcerated segment with irregular ulcers, prominent nodular mucosal elevations, marked friability, and luminal narrowing that prevented further advancement of the colonoscope. **b** Rectosigmoid colon, approximately 20 cm from the anal verge, showing another stricturing segment with ulceration, nodularity, and friable mucosa. **c**, **d** Representative colonic biopsy specimens demonstrating severe chronic active inflammation. **c** Dense lamina propria inflammatory infiltrates with crypt architectural distortion, cryptitis, and crypt abscess formation. **d** Focal crypt injury/rupture with prominent surrounding active inflammation.

Biopsies demonstrated severe chronic active inflammation with cryptitis, crypt abscesses, and granulation tissue (Fig. 2c, d). Tissue *M. tuberculosis* polymerase chain reaction and Fite-Faraco staining were negative for mycobacteria. Because pulmonary tuberculosis had been clinically diagnosed and concurrent ileocolitis was present, the intestinal findings were initially attributed to possible gastrointestinal tuberculosis, although IBD remained in the differential diagnosis.

### Persistent Disease and Diagnostic Reassessment

Despite completion of antituberculosis therapy, intestinal disease persisted. In contrast, repeat chest computed tomography performed approximately 6 months after initiation of therapy showed resolution of the previously noted noncalcified centrilobular nodules, consistent with a radiologic response of the pulmonary findings to antituberculosis therapy (Fig. 1b). Repeat colonoscopy after approximately 5 months of treatment showed segmental longitudinal ulcers with intervening pseudopolyps from the terminal ileum to the rectum (Fig. 3a–c). Histopathology demonstrated severe chronic active inflammation, tissue eosinophilia, and rare ill-defined non-necrotizing granulomas in the ileum, ascending colon, sigmoid colon, and rectum (Fig. 3d–f). Fungal and acid-fast stains were negative, and serologic testing for anti-*Saccharomyces cerevisiae* antibodies was negative.

**Fig. 3.**
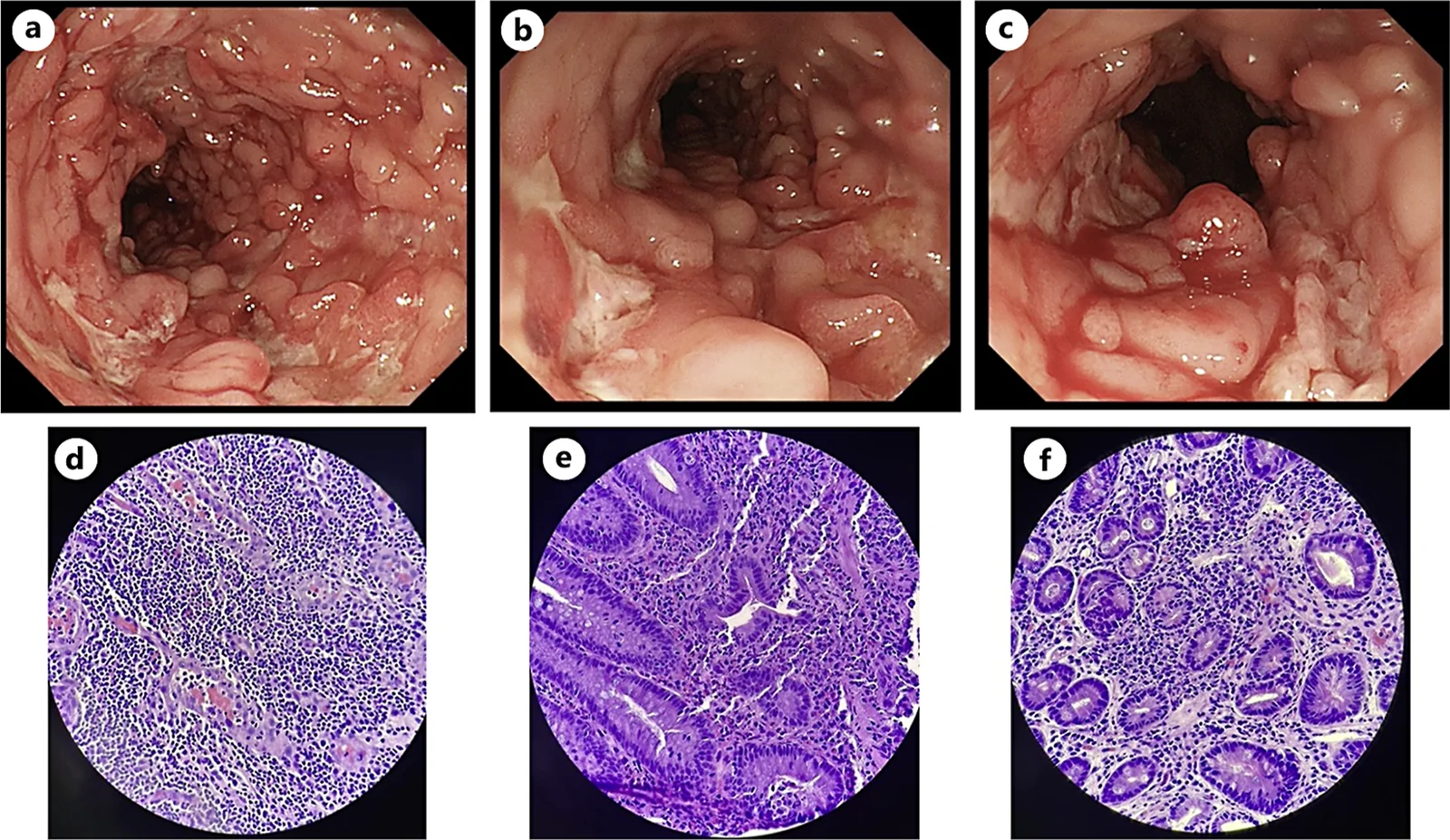
Endoscopic and histopathologic findings during diagnostic reassessment. **a–c** Repeat colonoscopy after approximately 5 months of antituberculosis therapy showing segmental longitudinal ulcers with intervening pseudopolyps forming a cobblestone appearance from the terminal ileum to the rectum: ascending colon (**a**), descending colon (**b**), and rectosigmoid colon (**c**). **d–f** Representative biopsy specimens showing severe chronic active inflammation with tissue eosinophilia and rare ill-defined non-necrotizing granulomas: granulomatous inflammation with prominent eosinophils (**d**), crypt architectural distortion with active cryptitis (**e**), and dense chronic inflammatory infiltrates with prominent eosinophilia (**f**).

The Crohn’s Disease Activity Index was 229, consistent with moderate-to-severe disease. The phenotype was classified as A2 L3 B2 by the Montreal classification, indicating ileocolonic stricturing CD. In view of persistent segmental disease despite a completed antituberculosis course, repeated negative intestinal microbiologic studies, longitudinal ulcers and skip areas, and chronic active colitis with crypt architectural injury, CD was favored over active gastrointestinal tuberculosis.

### Treatment and Follow-Up

The patient was started on oral mesalamine 2 g twice daily and ustekinumab. Subcutaneous ustekinumab 90 mg was subsequently administered every 8 weeks. On clinical follow-up, the intermittent abdominal pain and vomiting resolved, with improved oral intake and subsequent weight gain. The Crohn’s Disease Activity Index decreased to 45, consistent with clinical remission. Fecal calprotectin was 1,575 µg/g at baseline, increased to 1,740 µg/g during active disease, and decreased to 320 µg/g at week 16 of ustekinumab treatment.

Follow-up colonoscopy performed approximately 1 year after the preceding examination (week 48 of ustekinumab) demonstrated marked endoscopic improvement. The previously observed extensive longitudinal ulcerations and mucosal friability were no longer evident. Multiple post-inflammatory pseudopolyps remained throughout the colon, predominantly in the cecum and ascending colon, with smooth intervening mucosa and preservation of the vascular pattern (Fig. 4b–f). The ileocecal valve opening remained narrowed and prevented complete intubation of the terminal ileum despite repeated attempts using both a colonoscope and a gastroscope (Fig. 4a). The distal terminal ileum was visualized through the narrowed opening and biopsied.

**Fig. 4.**
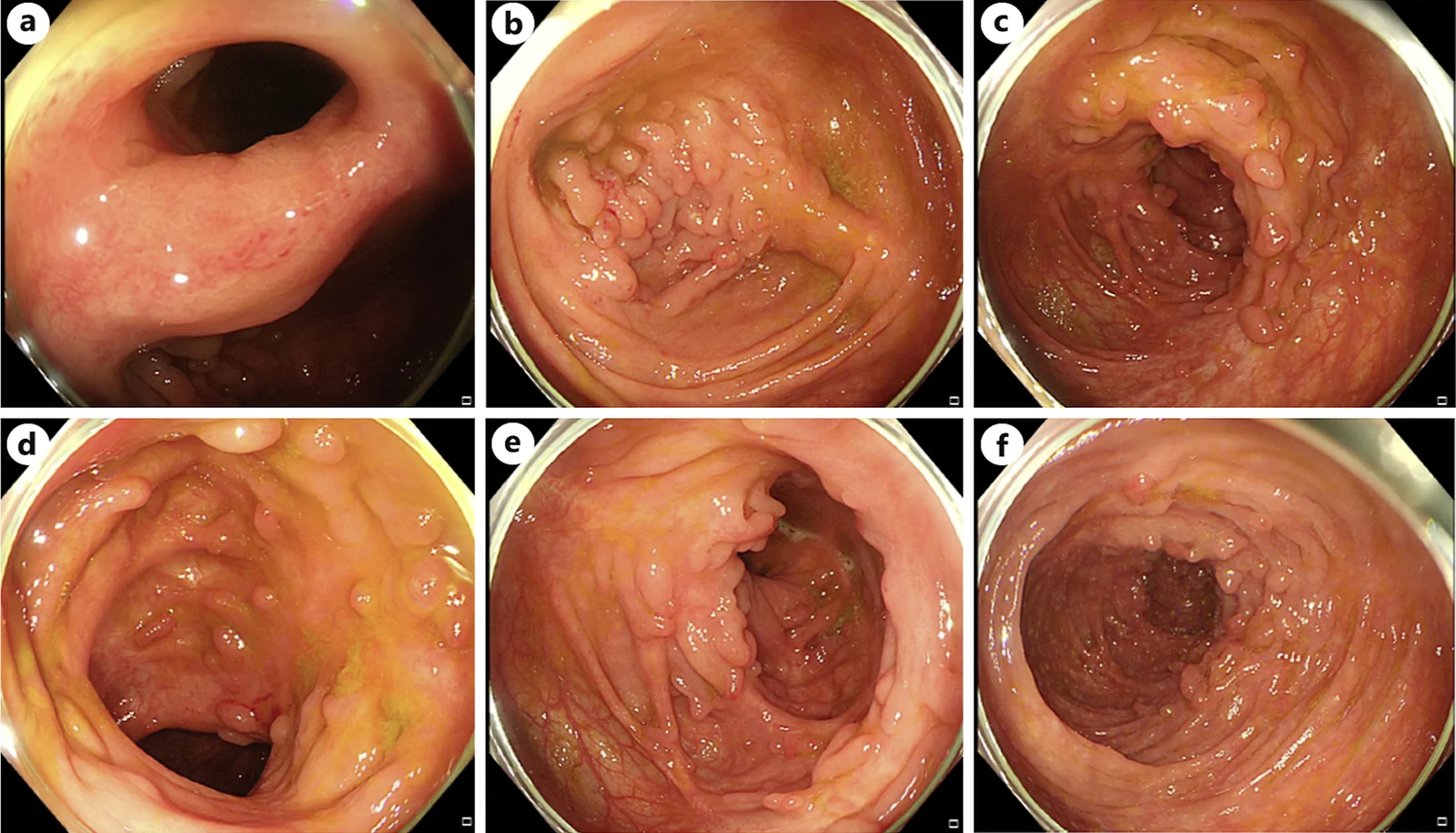
Repeat colonoscopic findings (week 48 of ustekinumab). Follow-up colonoscopy performed approximately 1 year after the previous examination (week 48 of ustekinumab) demonstrated marked improvement in the intestinal inflammatory burden. **a** Ileocecal valve showing persistent smooth narrowing with a small residual opening, preventing complete intubation of the terminal ileum. **b–f** Multiple post-inflammatory pseudopolyps with smooth, pink intervening mucosa and preservation of the vascular pattern: cecum (**b**), ascending colon (**c**), transverse colon (**d**), descending colon (**e**), and rectosigmoid colon (**f**). The previously observed extensive longitudinal ulcerations and diffuse mucosal friability were no longer evident.

Histopathologic examination showed focal mild active inflammation characterized by cryptitis in the cecum, without chronicity, granulomas, or dysplasia. Biopsies from the ascending, transverse, descending, and sigmoid colon and rectum showed no active inflammation or chronicity. The sampled terminal ileal mucosa also showed no diagnostic abnormality, active inflammation, villous blunting, granulomas, or dysplasia. These findings demonstrated substantial endoscopic and marked histologic improvement. However, persistent ileocecal valve narrowing, incomplete intubation of the terminal ileum, and focal residual cecal cryptitis precluded classification as complete endoscopic or histologic remission.

## Discussion

This case demonstrates how clinically diagnosed pulmonary tuberculosis may influence the attribution of concurrent intestinal inflammation to gastrointestinal tuberculosis in a tuberculosis-endemic setting. The Philippines remains among the countries with the largest tuberculosis burden; in 2024, it accounted for an estimated 6.8% of incident tuberculosis cases worldwide [6]. In this context, the patient’s pulmonary imaging findings and ileocolitis understandably led to initial treatment for possible gastrointestinal tuberculosis.

However, the intestinal course was inconsistent with an adequate response to antituberculosis therapy. Persistent segmental ileocolonic inflammation, repeatedly negative intestinal mycobacterial studies, and repeat colonoscopy showing longitudinal ulceration, pseudopolyps, and skip areas collectively favored CD. In contrast, transverse ulceration, a patulous ileocecal valve, and large, confluent, or necrotizing granulomas are more commonly associated with gastrointestinal tuberculosis [4, 7].

The diagnostic distinction is not absolute. Negative tissue mycobacterial tests do not fully exclude gastrointestinal tuberculosis, and antituberculosis treatment may alter intestinal findings [4]. Coexisting intestinal tuberculosis and CD also cannot be excluded with complete certainty. Nevertheless, the persistent Crohn’s-type endoscopic pattern, chronic active crypt injury, lack of intestinal response to antituberculosis therapy, and subsequent clinical and biochemical response to ustekinumab collectively supported management as CD.

Ustekinumab, an interleukin-12/23 antagonist, is an established option for moderate-to-severe CD [8]. Real-world Asian data also support its effectiveness and safety [9]. In patients with recent or treated tuberculosis, biologic initiation requires multidisciplinary review, documentation of adequate tuberculosis therapy, and careful surveillance for recurrence. In this patient, the reductions in Crohn’s Disease Activity Index and fecal calprotectin supported a clinical and biochemical response, although the residual fecal calprotectin elevation warranted objective endoscopic and histologic reassessment.

Follow-up colonoscopy approximately 1 year after the previous examination (week 48 of ustekinumab) demonstrated marked improvement in the intestinal inflammatory burden. The previously observed longitudinal ulcerations and diffuse mucosal friability were no longer evident. Residual post-inflammatory pseudopolyps were present throughout the colon, predominantly in the cecum and ascending colon, with smooth intervening mucosa and preserved vascular pattern. Histopathologic examination showed only focal mild active inflammation with cryptitis in the cecum, while biopsies from the remaining colonic segments and sampled distal terminal ileum showed no active inflammation, chronicity, granulomas, or dysplasia. These findings supported a substantial endoscopic and near-complete histologic response to CD -directed therapy. However, persistent narrowing of the ileocecal valve, incomplete intubation of the terminal ileum, and focal residual cecal inflammation precluded classification as complete mucosal healing or endoscopic remission.

The principal limitation of this report is the inability to prove or completely exclude prior gastrointestinal tuberculosis because the pulmonary diagnosis was clinically and radiologically established without microbiologic confirmation, while intestinal mycobacterial studies were repeatedly negative. Coexisting gastrointestinal tuberculosis and CD therefore cannot be excluded with absolute certainty. In addition, complete assessment of the terminal ileum during follow-up was limited by persistent narrowing of the ileocecal valve. Nevertheless, the longitudinal clinical, radiologic, microbiologic, endoscopic, and histopathologic findings, together with the objective response to ustekinumab, emphasize the importance of reconsidering an initial diagnosis of gastrointestinal tuberculosis when intestinal inflammation persists despite adequate antituberculosis therapy.

## Conclusion

Clinically diagnosed pulmonary tuberculosis may influence the interpretation of concurrent ileocolonic inflammation in favor of gastrointestinal tuberculosis, thereby complicating the diagnosis of CD. In patients with persistent or progressive intestinal disease despite an adequate antituberculosis course, repeatedly negative intestinal microbiologic studies and endoscopic and histopathologic findings favoring CD should prompt multidisciplinary diagnostic reassessment. Although clinical and biochemical improvement following CD-directed therapy may support the revised diagnosis, objective follow-up endoscopy is required before mucosal healing or endoscopic remission can be established.

## Acknowledgments

The authors thank Dr. Ian Homer Cua, Head of the Institute of Digestive and Liver Diseases, and Dr. Enrik John Aguila, Head of the Research Committee, for their guidance and support. A generative artificial intelligence tool was used for language editing and document formatting; all authors reviewed and verified the final content and remain fully responsible for the manuscript.

## Statement of Ethics

This report was prepared in accordance with the Declaration of Helsinki. Ethics approval was waived by the St. Luke’s Medical Center Institutional Ethics Review Committee. Written informed consent was obtained from the patient for publication of this case report and all accompanying images.

## Conflict of Interest Statement

The authors have no conflicts of interest to declare.

## Funding Sources

The article processing charge for this publication was supported by grants from the Institute of Digestive and Liver Diseases, St. Luke’s Medical Center – Global City, and the Research and Biotechnology Group of St. Luke’s Medical Center. The funding bodies had no role in the conduct of the study, preparation of the manuscript, or decision to publish.

## Author Contributions

J.P.D.P.: conceptualization, data curation, writing – original draft, and writing – review and editing. C.P.F.: clinical management, interpretation, and writing – review and editing. M.D.-F.: pulmonary management, data interpretation, and writing – review and editing. M.L.S.-J.: inflammatory bowel disease management, clinical interpretation, supervision, and writing – review and editing. All authors approved the final manuscript.

## Data Availability

The clinical data supporting this case report are not publicly available because of patient confidentiality but may be made available by the corresponding author upon reasonable request and subject to applicable institutional and ethical restrictions.
